# Benchmarks for Needed Psychiatric Beds for the United States: A Test of a Predictive Analytics Model

**DOI:** 10.3390/ijerph182212205

**Published:** 2021-11-20

**Authors:** Christopher G. Hudson

**Affiliations:** School of Social Work, Salem State University, Salem, MA 01970, USA; chudson@salemstate.edu

**Keywords:** psychiatric beds, inpatient care, benchmarks, psychiatric deinstitutionalization, predictive analytics, needs assessment

## Abstract

The ideal balanced mental health service system presupposes that planners can determine the need for various required services. The history of deinstitutionalization has shown that one of the most difficult such determinations involves the number of needed psychiatric beds for various localities. Historically, such assessments have been made on the basis of waiting and vacancy lists, expert estimates, or social indicator approaches that do not take into account local conditions. Specifically, this study aims to generate benchmarks or estimated rates of needed psychiatric beds for the 50 U.S. states by employing a predictive analytics methodology that uses nonlinear regression. Data used were secured primarily from the U.S. Census’ American Community Survey and from the Substance Abuse and Mental Health Administration. Key predictors used were indicators of community mental health (CMH) service coverage, mental health disability in the adult population, longevity from birth, and the percentage of the 15+ who were married in 2018. The model was then used to calculate predicted bed rates based on the ‘what-if’ assumption of an optimal level of CMH service availability. The final model revealed an overall rate of needed beds of 34.9 per 100,000 population, or between 28.1 and 41.7. In total, 32% of the states provide inpatient psychiatric care at a level less than the estimated need; 28% at a level in excess of the need; with the remainder at a level within 95% confidence limits of the estimated need. These projections are in the low range of prior estimates, ranging from 33.8 to 64.1 since the 1980s. The study demonstrates the possibility of using predictive analytics to generate individualized estimates for a variety of service modalities for a range of localities.

## 1. A Test of a Predictive Analytics Model

### 1.1. Introduction

One of the most enduring debates in mental health has been the wisdom of psychiatric deinstitutionalization. While some still question whether the dramatic reductions in psychiatric beds to date have been sufficient, many have suggested that some nations, such as the United States, have overshot the mark, that reductions have been precipitous, and that insufficient community mental health services have been developed [1]. Most commentators have increasingly argued that what is needed is a comprehensive and balanced system of mental health services that range from the most to the least restrictive [2,3]. Such arguments require that mental health planners accurately gauge the need for various service modalities, determining the optimal levels, whether for inpatient beds, day programs slots, supported housing units, or various outpatient treatment options. Just as empirically-based benchmarks for service outcomes are needed, benchmarks or guidelines for the levels of needed services are required for various localities. Unfortunately, most research methodologies currently being employed, including many quasi-experimental designs and random controlled trials (RCTs), fail to generate results that can support such planning decisions. An important alternative has involved the use of predictive analytic models [4] for generating estimates of needed service units, such as psychiatric hospital beds, that are locality specific. Among other advantages, predictive analytic models and the resulting tools can be used to estimate the simultaneous non-linear effects of various inputs and environmental conditions, thus enabling the discovery of optimal solutions to problems involving trade-offs in service deployment.

This study has aimed to employ one such model that was previously developed and tested on the international level [5] for the estimation of national benchmarks for needed psychiatric beds, for the development of similar benchmarks for the 50 U.S. states. It estimates a non-linear multivariate model of various levels of psychiatric disability throughout the U.S. for predicting the likely need in each state—whether it is more or less than what is currently provided—based in part on modeling the likely need under the counterfactual possibility of an optimal level of community mental health services. Thus, the current study seeks to both apply the model that was recently tested internationally and to adapt it for generating estimates of psychiatric beds needed on a state level in the United States. Selected paragraphs in the introduction and methodology have been adapted and condensed from [5]. 

### 1.2. Early Estimates, Pre-1975

Questions about the optimal level of psychiatric beds initially arose in the earliest years of deinstitutionalization, beginning with Tooth and Brook’s study in Britain [6], viewed by some as a classic. These researchers examined hospitalization statistics for 1954 to 1959 for England and Wales and concluded that 180 beds per 100,000 were needed at the time of their study, but extrapolated, on a linear basis, that the need would disappear by 1975. Many subsequent researchers criticized both their reliance on service levels, as well as the simple linear extrapolation methodology [7,8].

In the mid-70s, Lenora Bachrach [9] reviewed the then-existing research on this question for the U.S. National Institute of Mental Health (NIMH) report. Her focus was primarily on clarifying the many complex issues that need to be taken into account in developing such estimates, such as the differential rates of admission and length of stay, availability of community mental health services, philosophies of policy makers, and the like. In her annotated bibliography of this research, she reviewed 21 studies from 1964 to 1973. The earlier 12 of these studies from the 1960s proposed global estimates of bed need that ranged between 40 and 219 per 100,000, and with a mean of 83.8. In contrast, most of the later nine studies from the early 1970s generated estimates ranging from 20 to 375, with an average of 89.4. The vast majority of these relied on the assumption that actual hospitalization levels, which were high to begin with and then progressively lower, reflected the actual need. A number of the later studies during this period increasingly incorporated professional estimates. Several failed to describe any methodology. For these reasons, little confidence can be placed in these estimates, which are noted here primarily for their historical and contextual interest.

### 1.3. Assessment of Methodologies Used

Given the modest level of methodological rigor in the early research that Bachrach reviewed [9], it is not surprising that the next decade saw minimal interest in this problem, with one important exception. In 1986, Eric Gopelerud [10] reviewed seven newer estimation efforts undertaken by various U.S. mental health authorities in the early 1980s, with close attention to assessing the methodologies employed. These seven efforts, undertaken by four states, two national organizations, and one private insurance company, employed various combinations of methodologies: mainly the use of expert opinion, historical usage, and epidemiological and social indicators data. The generated projections from these studies ranged from 21.3 to 44.4 per 100,000 population, with a mean of 28.5. Gopelerud assumed—very questionably—that projected need levels should match actual utilization rates, and so compared these, which, with only two exceptions, failed to match. While the logic of this test leaves much to be desired, the most pertinent part of his analysis involved an ad hoc comparison of utilization with licensed beds levels. He hypothesized a non-linear relationship: that utilization should match the licensed bed availability, but only up to a point, after which, the highest rates of licensed beds should not produce further utilization. Five of the seven methods underestimated utilization; however, the formulas generated by the Michigan [11] and Massachusetts [12] projects were found to be the most accurate, providing projected rates of 35 and 44 per 100,000 population, respectively. Gopelerud’s analytical review, although limited by its over-reliance on historical utilization data, served to generate some pertinent comparative data on the results of the varying methodologies being used at that time. Gopelerud concludes his review by urging that “Multivariate statistical tools must be applied to larger sets of service areas to provide quantitative guidelines for planning services” [10] (p. 395).

### 1.4. Limitations of the Use of Hospital Utilization Data

Research on optimal hospitalization levels has been modest in scope and rigor, given many obvious problems that need to be surmounted. In contrast, research that documents these problems has been more extensive. Here, three studies related to one of these problems, the over-reliance on utilization data, will be briefly reviewed. While the hope of some has been that existing patterns of use reveal patterns of need, such an assumption ignores the many known limitations of such data. Patients may be inappropriately hospitalized, whereas others may be inappropriately not hospitalized. In addition, inappropriate hospitalization may be a result of inadequate community mental health, residential, and family supports, or, conversely, effective community services may prevent hospitalization for some, or, conversely, enable the needed hospitalization for others in the case of emergency services.

In a systematic review of the psychometric properties of instruments designed for the assessment of the appropriateness of existing hospitalization, McDonagh, Smith, and Goddard [13] found that one of the leading instruments, the Appropriateness Evaluation Protocol [AEP] [14], revealed that 29% of psychiatric hospitalizations were not needed or unduly prolonged, mostly typically due to the unavailability of community supports for those to be discharged. How many of those in the community, living on the street, or in jails and prisons require hospitalization has been a much more difficult question to answer. One recent systematic review [15], however, suggests that this number could be minimal.

There have been a variety of studies in recent years that have attempted to assess the effectiveness of community services, most typically assertive community treatment (ACT), in reducing hospital use. For example, Rosenheck and Neale [16] studied the adequacy of ACT services in the U.S. at nine Veteran’s Administration (VA) Hospitals over a five-year period and found significant and substantial reductions in hospitalization, most notably with those who were high hospital users to begin with. Similarly, Morrissey, Donner, and Cuddleback [17] found such reductions in a Washington state cohort of recipients of ACT services; however, such reductions were also only significant with the subgroup of the patients who were high users to begin with. While most such quasi-experimental studies cannot eliminate the possibility that such reductions are a result of either statistical regression or spontaneous remission, the results of both of these studies were in the direction expected and provide some support, though inconclusive, of such an effect. It may be that there is no single required intervention, but rather a package or system of community services—for example, outpatient, day, and residential programs—that is needed. Thus, such possibilities of the inappropriate use or avoidance of psychiatric hospitals cast serious doubt on the assumption that usage patterns translate into patterns of need.

### 1.5. Expert Opinion Research

Despite the many questions raised about the earlier uses of expert opinion as a means of determining optimal psychiatric bed levels, there has been a further study along these lines. This is the project undertaken by the Treatment Advocacy Center in 2008 [18], led by E. Torrey Wolfe, that has sought to employ an enhanced methodology for this purpose, one known as the Delphi technique. This involves the anonymous survey of a panel of known experts, and the iterative feedback of the results and thinking of the experts to their colleagues (without identities divulged) until a consensus has been achieved. In this project, 15 experts were identified from the ranks of mental health administrators, practitioners, and experts on psychiatric disorders. Each expert was asked to propose an optimal rate of psychiatric hospitalization; one that assumes “good outpatient programs and availability of outpatient commitment”. The process resulted in a mean estimate of 50 beds per 100,000, with a range of 40 to 60. The report on this study pointed out that 42 of the 50 U.S. states provided less than half of this minimum benchmark number. It also discussed various causes and manifestations of this gap; for example, the growing problems of seriously mentally ill individuals stuck in emergency rooms with no hospital bed to be referred to. Despite the face validity that such an estimate may have, like earlier ones, it must be treated as provisional, given the many known limitations of expert opinion studies. In this case, it is unknown how familiar these experts were with the many earlier estimates discussed in this review. Another important limitation is that this figure is proposed as a global estimate, with no allowances made for varying levels of population need and community supports in various localities.

### 1.6. Recent Modeling Strategies

In 2016 North Carolina researchers reported on the results of a study using an alternative modeling methodology to examine the relationship between the utilization of inpatient psychiatric care and the waiting times in emergency rooms, a problem identified earlier by the TAC study [18]. The study, led by Elizabeth La and her associates [19], examined patterns of patient flow through psychiatric facilities in a region of North Carolina. They used a discrete events simulation model that was able to reproduce the actual emergency room waiting times for beds, which stood at a mean of 3.5 days. The model permitted the researchers to examine the likely effects of adding varying numbers of beds, with the goal of reducing emergency room waiting times to less than a day. It was only the most ambitious scenario examined, one involving increasing the bed capacity by 165%, that promised to reduce waiting times to under one day. Such an increase of 365 additional beds, designed to achieve the lowered wait times, would thus require an increase in the coverage rate in the region from 28 to 39 beds per 100,000 population. However, basing the provision of services on the size of waiting lists, and simply attempting to optimize a single such indicator of need, ignores the many variables that are unrelated to the need for psychiatric treatment and that are widely believed to determine the size of waiting lists. For example, there is no specification of the number of patients recommended for hospitalization who refuse, and, thus, who were not placed on the lists. As a result of such considerations, the rate of 39 must be regarded as a low-end estimate.

The North Carolina study represents an example of research which has sought to estimate hospital bed need based on the undesired effects of the underutilization of hospital care. O’Reilly, Allison, and Bastiampiallai proposed another such methodology: a “naturalistic approach that estimates the required number of psychiatric beds by comparing the bed levels at which negative outcomes develop in different jurisdictions.” [20] (p. 1). It is essentially an incremental approach that attempts to adjust hospital bed deployment until unmet need in the community is eliminated. The strategy is not dissimilar to several described in the literature, such as La et al., that attempt to identify optimal bed utilization rates, based on the notion that excessively low vacancy rates indicate unmet bed needs. Central to many of these calculations is the use of the Erlang equation, which links beds numbers with average occupancy and turn-away, i.e., the proportion of times a bed is not available for the next arriving patient. A research review by Rodney Jones in the UK has concluded from such analyses that,

“psychiatric hospitals with fewer than 100 beds should be operating below 85% average occupancy, whereas larger hospitals should be limited to a maximum of 85% occupancy in order to protect both patients and staff from untoward incidents arising from busyness”. [21] (p. 1).

Each of these approaches are limited by their assumption that unmet need necessarily translates into an increased demand for beds, ignoring the possibility of severely disturbed individuals who require hospitalization but do not ask nor are referred for it. In addition, such approaches do not permit an assessment of the over-deployment of hospital care. They often require a wider array of data indicators for the consequences of unmet need than are ordinarily available, and thus are very difficult to use, especially on a multi-locality basis.

An alternative strategy for estimating needed hospital beds was tested by Davis and Lowell [22], who employed a complex systems framework, along with artificial neural networks, to model the optimal number of beds for various types of care in the state of Maine. Their approach employs the Gutenberg–Richter power law and the concept of self-organized criticality, which states that the severity of an event, whether an earthquake, the collapse of a sand pile, or the occurrence of many life events, such as hospitalizations, will be inversely proportional to their frequency. While the logic of their analyses and the plausibility of its underlying assumptions fall short, their results for the mental health system in Maine do closely match their theoretical predictions. Extrapolating these results to the United States as a whole, they conclude that,

“given that the United States has a population of 260 million, and using our SH [state hospital] phase heuristic of 22 beds per 100,000 of the general population, our predicted number of SH beds is (2600) (22) = 57,200. This is fairly close to the 58,510 beds that are actually staffed at the end of the fiscal year 1995 according to the National Association of State Mental Health Program Director’ Web Page”. [22] (p. 226).

Despite its limitations, Davis and Lowell’s modeling strategy shows promise, especially as it attempts to both model the optimal trade-offs between various types of mental health care and predict needed hospital beds and other units of care. This methodology will require considerable further testing in a variety of localities in the U.S. and beyond.

One of the most recent initiatives for estimating needed psychiatric hospital beds was undertaken by Hudson [5], who employed a predictive analytic modeling strategy that tested for nonlinear relationships in order to generate estimates for 166 nations. Variables employed in the final model included psychiatric disability measured by life years lost; percentage of married adults; life expectancy; and service data on inpatient care and community mental health from the World Health Organization. The final model estimated accounted for 42.3% of variance in levels of psychiatric hospitalization levels. This model was then used to project both actual hospitalization levels, and, most importantly, psychiatric hospitalization levels given the counterfactual condition of a very high level of community mental health services, set at a level equivalent to the top 10% of national CMH care systems. This second set of projections was then compared with reported levels of hospitalization in each nation. It revealed that 69.6% of nations had significantly lower rates of beds than needed; 18.3% had levels commensurate with need; and 11.8% had significantly elevated rates of inpatient care. Those that were low included the United States, the United Kingdom, and Australia, those that were moderate included various EU nations, and those that were very high included Japan, Korea, and the Czech Republic. The overall rate of needed beds projected was 45.5 per 100,000 population, similar to other recent studies, such as Torrey et al., [18] at 50, and La et al. (2016), at 39. While this study had the advantage of producing model estimates tailored to diverse national environments, and was commensurate with those independently produced by other estimation strategies, considerably more work is needed, not only on validation, but in producing more fine-gained estimates for various modalities of inpatient and outpatient care in local sub-national environments.

### 1.7. Summary and Research Questions

Over 60 years of sporadic work on the problem of determining benchmarks for levels of psychiatric beds has seen only modest progress. The progression of continually revised results, in part, reflects a frequent over-reliance on the use of existing hospitalization statistics, expert opinion, and the analysis of single systems of care. Gaps in the existing body of research include the comparison of multiple systems, use of psychiatric disability data on the general population, adjustment for availability of community mental health services and other social supports, and the use of modeling to generate individualized system estimates. Nonetheless, prior research has begun to address several of these gaps. Most recently, the Hudson [5] study has completed preliminary testing of a predictive analytics model that shows promise in generating individualized national estimates of need using a predictive analytics model with disability, CMH service, and sociodemographic data as indicators. The current study aims to test and build on this model on localities within a single nation (the U.S.) and to revise it as needed based on this nation’s unique conditions. Specifically, this study is designed to answer the following questions: (i) Can variation in psychiatric hospitalization rates in the United States be explained based on levels of disability, community mental health care, and socio-demographic indicators of social development and social support? (ii) What are the rates of needed inpatient psychiatric care that can be projected for each state for which there is available data? Which states and how many exceed, meet, or fall short of such potential benchmarked levels of need?

## 2. Materials and Methods

### 2.1. Overview

This project employs a secondary analysis of publicly available data sources, specifically one that uses a predictive analytics strategy with non-linear regression modeling for the estimation of both actual and needed levels of psychiatric hospitalization for the 50 U.S. states. The model is then used to estimate levels of bed need under the counterfactual condition involving an optimal level of community mental health services. The approach replicates and builds on previous research, which has demonstrated that levels of psychiatric hospitalization are moderately to strongly correlated with levels of psychiatric need, socioeconomic indicators, and levels of community mental health services available, and is able to project levels of bed need using a predictive analytics model that has been partially validated [5,23,24].

### 2.2. Variables and Data Sources

Variables for this study were selected to be maximally parallel with those used in the preceding [5] study. The dependent variable is the rate of psychiatric inpatient beds provided in each of the 50 states. The figures used include both private and public state facilities, and acute and long-term care. These facilities range from private inpatient psychiatric units in community hospitals to specialty psychiatric hospitals operated by state governments. Statistics for these were downloaded from a report on the *2018 National Mental Health Services Survey*, conducted by the Substance Abuse and Mental Health Services Administration of the U.S. government [25] (Table 4.7, pp. 125–126). Each was converted to a population rate (per 100,000).

Indicators of the availability of community mental health services—key predictors for the study—were also downloaded from the same SAMSHA report as cited above. These are the rates of patients receiving outpatient mental health services, the number of residential mental health beds, and the number of day programs (including partial hospitalization) programs in each state. Each of these three indicators were converted to population rates (per 100,000), transformed into z-scores, and averaged to create a composite index of community mental health services availability.

Another predictor used in this study is the overall rate of mental health disability in each state. Statistics of these were downloaded from the American Community Survey [26] for 2018, conducted by the U.S. Census Bureau. Specifically, numbers of disabled individuals were downloaded for various age groups, summed, and then divided into the total population in order to generate a percentage rate for each geographic area. A question was used from the American Community Survey involving the presence of a mental disability, as perceived by the respondent, in each of the family members inquired about. Specifically, the census enumerator asks about the presence of a physical, mental, or emotional condition lasting six months or more involving a difficulty in learning, remembering, or concentrating. Due to the fact that this is broader than many traditional definitions of mental disability that have focused on mental illness, its validity was further investigated using other available indicators of mental illness. The item was found [23] to have a strong correlation with two items, both on the county level, from the Center for Disease Control’s (CDC’s) behavioral health survey: (i) Number of mentally unhealthy days in the previous month (*r* = 0.725; *p* > 0.001), and (ii) Number of days the respondent reported frequent mental distress (*r* = 0.743; *p* > 0.001).

Two control measures of the varying demographic conditions in each state were mean expected longevity from birth in years, and percentage of adults over 15 who were married in 2018. Whereas longevity is treated as a proxy indicator of the level of socio-economic development [27], marital status is used as a proxy for social support [28]. These two indicators were computed from data that were downloaded from the American Community Survey [26] for 2018, conducted by the U.S. Census Bureau. A number of additional indicators of socio-economic conditions for each state were also tested, such as an index of liberalism, but none of these improved the model’s predictability of psychiatric hospitalization rates.

### 2.3. Sampling

All 50 U.S. states were used in this study, excluding any territories and the District of Columbia. For any analyses involving aggregated statistical measures, such as means and regression coefficients, each state was weighted by its relative population for the 2018 year.

### 2.4. Analysis

All analyses were undertaken with a predictive analytics strategy that employs regression modeling to generate a statistical model, one which is then used for predictive purposes. At each stage, SPSS Statistics Version 27.0 was used. Preliminary analyses were conducted using basic univariate and bivariate descriptive statistics. These included an examination of a variety of predictors not used in the final modeling, most typically excluded due to multicollinearity, as indicated by correlations of 0.80 and higher. The form of the bivariate correlations of the predictors with the dependent variable, rate of hospitalization, was examined through scatter plots and a nonlinear regression analysis using quadratic and cubic terms. Due to the fact that the distribution of hospitalization rates deviated significantly from normality, these were normalized using an inverse transformation (1/bed rate), resulting in a corrected kurtosis of −0.015 (SE = 0.657) and skewness of −0.256 (SE = 0.334). Those for which significant nonlinearities were detected using scattergrams had squared and/or cubic terms computed for use in the multivariate modeling.

Psychiatric hospitalization rates (per 100,000 population) were regressed on the various predictors chosen for the model. These predictors were initially chosen based on the prior study, with efforts made to introduce new predictors to improve on the model’s predictability. Some nonsignificant predictors were retained in the final model for several reasons: to enhance comparability with the international model being replicated and the total population of all 50 states being modeled, the need for inferential tests for the generalizability of the coefficients was therefore minimized. In addition, because the computation of the projections permitted the calculation of confidence intervals for the estimates for individual states, the need for an overall aggregate significance of the coefficients was further minimized.

Once a final model was estimated, national estimates and their 95% confidence intervals (CIs) were computed for needed psychiatric hospitalization based on psychiatric disability, CMH services available, percentage married, and longevity. An adjusted estimate was also computed using the mean levels of community mental health services for the 10% or five states from the top decile for community mental health services. This rate was then substituted into the equation for the estimated model, with the estimated rate of needed psychiatric hospitalization and associated confidence intervals recomputed. Finally, regional means for the adjusted rates and their 95% confidence intervals (CIs) were computed, along with a categorical variable indicating the positive or negative gap between actual hospitalization rate and estimated need in three groups: (i) those states for which actual hospitalization rates were less than the lower 95% CI of need; (ii) those for which the actual rates fell within the confidence interval of the need estimate; (iii) and those for which available hospitalization rates exceeded the upper CI of the need estimate. Finally, the aggregate rates computed were compared with the results of both the previous model’s projection for the U.S. as a whole, as well as with projections of those of other researchers.

### 2.5. Limitations

As a predictive analytic estimation study, its primary limitations involve a degree of uncertainty that originates from several sources. Results on the reliability and validity of the various predictors, as well as the hospitalization rates, have not been sufficiently established by previous research. In addition, the final model left over three-quarters (78.6%) of the variation in hospitalization rates unexplained. For this reason, it was essential that confidence intervals be included for the model’s estimates in order to permit an assessment of the range within which estimates can be reasonably made; that is, within 95% confidence interval limits. These also permit an assessment of the congruence of the estimates with those from previously published studies in order to provide evidence for the validation of the estimates of the current study.

## 3. Results

### 3.1. Psychiatric Hospitalization Rates

In 2018, the mean population rate for beds in mental hospitals in the United States, based on the SAMSHA data, was 39.0 per 100,000, with a median of 31.4. This rate varied dramatically, from 18.2 in Nevada to a high of 126.7 in Louisiana, with the middle 50% of states falling within the 25.0 to 42.2 range. A more accurate set of statistics on the access of the U.S. population to mental hospital beds is revealed when these rates are weighted for relative state population. The overall weighted mean is 39.1, with a median of 32.5. The lowest regional levels are found in the East North Central States (29.4), whereas the highest levels occurred in the Mountain States (79.0). While such numbers reveal the relative and absolute rates, in order to determine which of these may be low or high relative to need is a task that the remainder of this analysis will now turn to.

### 3.2. Predictors

The aggregate rate of mental disability among adults in 2015, using the ACS data, was found to be 5.26%. This is largely consistent with the rate of serious mental illnesses found in previous research, at 5.3% [23]. The mean z-score for the community mental health services index for the various states was −0.2813, with the minimum at −1.349 and maximum at 2.070. The percentage of adults, 15 and older, who were married stood at 38.4%, whereas the life expectancy, from birth in years, was found to be 79.15 years in the year of interest, 2018.

### 3.3. Modeling Psychiatric Hospitalization Rates

A core questions of the study is whether the variation in psychiatric hospitalization rates in the United States can be explained, at least partially, based on the levels of psychiatric disability, community mental health care, and other demographic measures of mental health’s sociocultural context. This was undertaken in this study by regressing the normalized state psychiatric hospitalization rates on the study predictors. After testing several exploratory models, it was found that none of these preliminary models improved the overall predictability of the hypothesized model based on the prior international study, involving psychiatric disability, community mental health, longevity, and the percentage of adults married. The final model accounted for 21.4% of variation at a significant overall level (*R^2^* Adj = 0.214; F = 2.9935; *p* = 0.013). As a result of the inverse normalization of the hospitalization rates that was used, each of the coefficients must be interpreted as having the opposite signed direction. Most notably, the effect of psychiatric disability was found to have a standardized beta coefficient of −1.676 and a partial r of −0.175, which represents an expected *positive* relationship: the more disability in the population, the more psychiatric hospitalization.

The most substantively strong effect is that of the percentage of married adults, a proxy measure for potential social support, which was found to have a beta coefficient of 0.349, with a partial r of 0.375, indicating that the higher the percentage of married adults, the lower the hospitalization rate. Regarding the life expectancy, a proxy measure of socioeconomic development, a positive effect was found, as there was a beta of −0.349 and a partial *r* of −0.245. Consistent with the international study, this indicates that the higher the average life expectancy, the greater the level of psychiatric hospitalization. Since the community mental health services index had to be disaggregated to its linear, squared, and cubic components, its effect on psychiatric hospitalization was found to be more complex, with its linear and squared components having a negative effect, reducing hospitalization, and its cubic component, a positive effect, in aggregate, minimizing hospitalization. This is after a correction for the inverse normalization is made. Since several of the T values for the regression coefficient did not attain the level of 0.05 significance, these results must be read with considerable caution, indicating that the international model tested has been only partially replicated here. Both the regression coefficient and the overall regression statistics for both the original international model and the current study model are reported in Table 1.

The second question of the study asks about the rates of needed inpatient psychiatric care that can be projected for each state for which there is available data. This can be investigated since an explanatory model has been estimated that significantly accounts for a meaningful proportion of the variation in hospitalization rates among the states. This is achieved by the use of each of the unstandardized regression coefficients in computing a weighted average or projection for each state with an important exception (correcting or reversing for the inverse normalization used). Instead of using the actual value for a state’s level of community mental health provision, a counterfactual or ‘what if’ version of this value is instead used: one that represents an optimal level of community mental health services. The specific value used was set at a level equivalent to that of the 90th percentile of the states, or 1.0206 for the CMH index. This is based on the assumption that such an optimal level will mitigate the need for inpatient psychiatric care.

Table 2 summarizes the results of the computation of the projections for each of the 50 U.S. states. The second and third columns in this table involving the psychiatric hospitalization rate and the model estimates of needed beds unadjusted are included for comparison purposes. The third column reports the primary estimates, adjusted for an optimal level of community mental health services. The final three columns provide the 95% confidence intervals for the adjusted estimates and a summary comparison of the adjusted estimates with the actual rates of provision. If the actual rate falls within the confidence intervals, it is assumed that the actual rate approximates the needed level, and likewise, ones below or above these levels represent states of insufficient or excess hospital provision.

In total, almost a third of the states, or 16 or 32%, have levels of psychiatric hospital provision that are significantly less than what is projected as needed. Noted examples are Nevada, Georgia, and Hawaii. A slightly smaller proportion of the states, or 14 or 28%, provide inpatient care at a level above what is projected as needed. Examples include Colorado, Louisiana, and Arizona. The remaining states, 20 or 40%, provide inpatient care at a level that is commensurate with the projected need, and these include New York, Illinois, and Massachusetts. In aggregate, the model permits a projected level of needed psychiatric beds at 34.88 per 100,000 population, which is slightly above an unweighted mean rate of 32.89. These levels ranged from a low of 24.66 per 100,000 population in Wyoming to a high of 49.45 in New York.

Table 3 summarizes these results when broken down by regions and divisions as defined by the U.S. Census Bureau. Though all four regions—Northeast, Midwest, South, and West—have psychiatric hospitalization levels that are, in aggregate, commensurate with the projected need, of the nine divisions of the states, three of them—the West North Central, South Atlantic, and West South Central—have hospitalization levels greater than needed, and only one region—the Pacific (California, Oregon, Washington)—has an overall hospitalization rate that is less than what is projected as needed.

Finally, the face validity of the overall estimate for the U.S. was examined by comparing the aggregate rate of psychiatric bed need with rates found in other related studies. Table 4 presents these results, showing that the overall projected rate for the U.S. of 34.5 per 100,000 is in the low range of the results from other U.S. studies conducted since the 1980s. While this project’s projected rate is only slightly higher than the Goperud estimate [10] of 33.8 that was based on six studies, it is less than the Treatment Advocacy (2008) expert consensus estimate of 50, the La et al. [19] North Carolina study with a result of 39, and the 64.1 rate projected for the U.S. from the predecessor international study [5], based on 2015 data. It should be noted that the earlier historical rates of the projected need from the 1950s through the 1970s, which ranged from 83.8 to 180, were based largely on actual hospitalization rates, obtained either very early in or prior to the start of psychiatric deinstitutionalization, and, as such, are a less relevant basis for a comparison with the more recent projected levels.

## 4. Discussion

This study confirms that the level of need for psychiatric beds throughout the United States is commensurate, though somewhat lower than other recent studies have suggested. Specifically, it reveals that the United States requires around 34.88 beds, or between 28.06 and 41.70 per 100,000. This is slightly less than the 39.10 being provided, and less than several earlier projections, which ranged from 33.8 to 64.1. An important feature of the model that was used to make these estimates is that it provides a basis for individualized estimates for each of the 50 states, revealing a moderate level of diversity, ranging from a low of 24.66 in Wyoming to a high of 49.45 in New York. In total, 16 or 32% of the states provide inpatient psychiatric care at a level less than estimated need; 14 or 28% at a level in excess of need; with the remainder at a level within the range of estimated need.

This study has sought to replicate a model previously developed and tested on the international level with 166 nations, using parallel predictors. For the U.S., this estimated model proved less predictive than expected, though it still accounted for a meaningful proportion of variation among the geographic units included. At the same time, the aggregate estimate for the United States has revealed a level of service provision that is considerably closer to the estimated need than the prior international study, which suggested that the need was substantially greater than the actual beds provided, or 23.6 beds per 100,000 compared with the estimated need of 64.1 for the U.S. Given the reality that the predictors used, as well as the state figures for psychiatric beds, were from different reporting systems, using somewhat different definitions, it is not unexpected that there should be such a divergence of findings. Some of the differences between the 2014 estimates and those from the current study, conducted with 2018 data, could of course be due to changes in conditions over these four years. These could include changes in the economic and demographic conditions over these four years, as well as mental health system development, but it would be unlikely that these would account for such a substantive change. When the original international study was undertaken, it was expected that each country would need to use its own data to generate estimates for its local mental health authorities. Perhaps only when commonly agreed upon metrics are available, not only between nations, but within nations, will it be possible to use an identical model for an estimation of need and other mental health system parameters.

It is unlikely that the noted changes in the levels of psychiatric bed need in the United States is due to declines in the absolute level of psychiatric disability in the various state populations examined. The primary reason for this assumption is that there have been negligible changes in the level of mental health disability in the primary indicator used since earlier studies, or 0.052 in this 2015 study and 0.053 based on a study [23] based on parallel data for 2007. Though it is possible that improvements in community mental health coverage could account for this change, there is little data that would suggest such an explanation. In fact, improvements in such services could very well contribute to more hospitalization through enhancements in emergency care and other referral services. The two sociodemographic predictors used in this study had a similarly powerful effect in this study as in the earlier international study, revealing that social supports provided by marriage again proved to minimize the need for hospitalization, and the more favorable socioeconomic conditions revealed by the proxy variable of longevity continued to suppress the hospitalization rates. The effect of each of these variables no doubt reveals only the ‘tip of the iceberg’ of complex causal relationships involving multiple variables, but are nonetheless consistent with much prior research. Preliminary exploratory analyses investigated the possibility that the average liberalism in the various states could have minimized mental health stigma and thus led to more community care and less need for hospitalization, but no such effect could be detected.

As a predictive analytic estimation project, limitations of this study involve an uncertainty that originates from several sources. Results are dependent on the reliability and validity of the various predictors, as well as the hospitalization rates, which have not been fully established. In addition, the final model accounted for only a third (32.5%) of the variation in hospitalization rates. For this reason, it was essential that confidence limits, based on statistical significance, be included for the projections to permit an assessment of the range within which these estimates can be reasonably made; that is, with 95% percent confidence. These CIs also permit an assessment of the congruence of the estimates with those from previously published studies in order to provide evidence for the validation of the estimates of the current study.

Strengths of this study are not only the individualized state estimates and an assessment of their confidence levels, but also the partial or preliminary validation of results with those from the previous research, as imperfect as it has been. The advantage of 95% percent confidence intervals is that they should encourage individual state mental health authorities to use a variety of other local data sources, both qualitative and quantitative, in refining their own estimates of the current and projected need. The model permits an investigation of various ‘what-if’ questions; for example, ones involving the impact of alternative levels of community mental health service deployment or improved socioeconomic conditions. As is the case with any benchmarks, these should only be understood as general guidelines to be used alongside a variety of other need and service indicators.

## 5. Conclusions

There are several conclusions and implications that can be drawn from this research that depend on the particular type of service and locality under consideration. In general, this study enables state mental health planning authorities in the United States to seriously consider operational goals for either increasing, maintaining, or decreasing psychiatric beds, and, in some cases, for redeploying resources to community mental health services. It provides sufficient data to begin to determine how to ‘right-size’ and balance the segment of a state’s mental health system involving inpatient services. It also demonstrates the possibilities for similar studies for other modalities of mental health care, whether outpatient clinics, day programs, ACT programs, or supported residential or employment programs. Separate studies that compare various types and models of mental hospitalization also need to be conducted; most notably, acute care in general hospitals versus care in specialty public or private psychiatric hospitals. Furthermore, larger states with regional, county, or municipal mental health authorities would best refine the methodology used in this study, using their own local data to determine which of such localities merit an increase or decrease in the inpatient services offered, with the aim of enhancing the coherence, responsiveness, and effectiveness of the state’s entire mental health system.

## Figures and Tables

**Table 1:** Regression statistics for model of variations in psychiatric inpatient care (*n* = 50).

	International Study, 2015 (166 Nations)				Current U.S. Study (51 States and DC)			
	B	BETA	T	Partial r	B	BETA	T	Partial r
REGRESSION COEFFICIENTS								
Intercept	356.205		6.718		0.073		0.549	
Disability Rate, Total	−23028.38	−3.338	−5.631	−0.388	1.905	1.456	1.013	0.153
Disability Rate Sq	424811.3	3.36	5.809	0.399	−20.26	−1.676	−1.165	−0.175
C Community Mental								
Health Mean, (CMH)								
Z score	31.687	0.667	3.623	0.262	0.001	0.038	0.156	0.024
CMH Score Squared	−12.402	−1.616	−3.281	−0.239	0.005	0.273	1.598	0.237
CMH Score Cubed	1.114	1.192	3.139	0.229	−0.004	−0.403	−1.580	−0.235
Adults Married, %	−1.403	−0.308	−4.652	−0.329	0.002	0.349	2.642 *	0.375
Life Expectancy	73.519	0.321	3.989	0.0286	−0.002	−0.349	−1.655	-0.245
EQUATION								
STATISTICS								
R			0.667				0.57	
R2			0.444				0.325	
Adj. R2			0.423				0.214	
DF, Regression			178				43	
DF, Residual			7				7	
F			20.389				2.936	
P			0.000				0.013	
Mean Cook Distance			0.009				0.007	
Mean Standardized Residual			0.000				0.000	

NOTES: Data weighted by relative population size. For the U.S., 2018 total state psychiatric beds per 100,000 expressed as normalized variable using inverse transformation (1/bed rate) (kurtosis: skewness); because of this, in the U.S., negative coefficients represent positive associations and vice versa; * *p* < 0.05.

**Table 2:** Psychiatric mean hospitalization rates for U.S. states (per 100,000 pop.), model predictions, and adjusted model estimates.

State	Psych. Hosp., Beds per 100,000	Model Estimates of Needed Beds, Unadjusted	Model Estimates, Adjusted for Top 10% CMH	95% Confidence Limits for Adj. Model Estimates		Comparison Actual and Needed (Adj.) Psychiatric Hospital Beds ^1^
Colorado	114.3	32.74	31.44	25.67	37.2	Actual > Needed
Louisiana	126.68	34.05	40.63	29.99	51.28	Actual > Needed
Arizona	110.47	38.29	35.99	30.72	41.27	Actual > Needed
Missouri	82.49	30.58	29.21	25.35	33.08	Actual > Needed
Montana	53.89	26.46	26.06	20.39	31.73	Actual > Needed
Florida	69.8	36.68	38.91	32.35	45.48	Actual > Needed
Connecticut	59.1	72.37	37.46	19.69	55.23	Actual > Needed
Alaska	46.52	30.55	30.32	24.9	35.73	Actual > Needed
Kansas	38.51	26.45	25.33	21.65	29	Actual > Needed
Arkansas	48.25	31.78	31.76	23.75	39.78	Actual > Needed
Wyoming	36.63	25.34	24.66	20.68	28.64	Actual > Needed
North Dakota	38.45	29.62	28.39	22.73	34.05	Actual > Needed
Delaware	38.72	26.27	31.65	27.12	36.17	Actual > Needed
Utah	28.45	23.58	23.6	18.35	28.86	Actual = Needed (w/95%)
New Jersey	42.23	35.96	35.1	27.23	42.98	Actual = Needed (w/95%)
Nebraska	30.97	26.98	26.23	21.84	30.62	Actual > Needed
West Virginia	45.87	42.9	40.82	22.77	58.86	Actual = Needed (w/95%)
Indiana	30.9	29.06	27.68	23.81	31.54	Actual = Needed (w/95%)
Pennsylvania	35.76	33.01	32.65	27.05	38.24	Actual = Needed (w/95%)
Virginia	32.58	29.83	30	26.08	33.91	Actual = Needed (w/95%)
Alabama	33.65	33.66	31.84	25.97	37.72	Actual = Needed (w/95%)
Mississippi	38.71	39.07	38.15	25.8	50.49	Actual = Needed (w/95%)
Ohio	28.83	30.55	30.03	25.11	34.95	Actual = Needed (w/95%)
Tennessee	27.72	30.72	29.17	24.99	33.34	Actual = Needed (w/95%)
Iowa	24.18	27.38	26.15	22.1	30.2	Actual = Needed (w/95%)
Illinois	31.27	32.54	34.27	27.18	41.36	Actual = Needed (w/95%)
Michigan	29.83	35.18	33.4	28.51	38.28	Actual = Needed (w/95%)
Vermont	30.93	37.76	35.17	24.16	46.19	Actual = Needed (w/95%)
New York	42.7	52.22	49.45	36.29	62.6	Actual = Needed (w/95%)
Wisconsin	24.35	29.53	28.62	24.64	32.6	Actual < Needed
North Carolina	25.05	27.6	29.57	25.83	33.31	Actual < Needed
Maine	31.39	37.3	37.32	25.1	49.54	Actual = Needed (w/95%)
Rhode Island	41.35	51.97	49.42	34.85	63.99	Actual = Needed (w/95%)
South Carolina	26.24	30.75	31.48	26.74	36.22	Actual < Needed
Kentucky	29.61	38.22	35.9	24.42	47.37	Actual = Needed (w/95%)
Massachusetts	32.62	40.5	39.55	31.44	47.66	Actual = Needed (w/95%)
Texas	24.66	23.45	30.28	25.12	35.43	Actual < Needed
Washington	24.73	32.58	30.89	25.12	36.66	Actual < Needed
Idaho	19.7	23.96	24.86	19.48	30.24	Actual = Needed (w/95%)
Minnesota	23.23	30.96	29.43	24.32	34.54	Actual < Needed
Oklahoma	21.33	26	27.18	23.12	31.24	Actual < Needed
Maryland	26.6	35.49	34.53	28.19	40.87	Actual < Needed
California	33.3	34.92	43.54	35.03	52.06	Actual < Needed
New Hampshire	20.37	28.18	26.86	22.71	31	Actual < Needed
New Mexico	31.95	40.78	43.29	33.25	53.33	Actual < Needed
South Dakota	19.1	27.29	26.5	22.25	30.75	Actual < Needed
Oregon	25.27	38.32	35.98	27.67	44.29	Actual < Needed
Hawaii	24.3	23.47	37.16	24.63	49.69	Actual < Needed
Georgia	18.89	27.4	31.46	26.58	36.33	Actual < Needed
Nevada	18.15	28.03	35.41	28.76	42.07	Actual < Needed
Mean (Unweighted)	**38.81**	**33.17**	**32.89**	**25.83**	**39.96**	
Mean (Weighted)	**39.1**	**33.64**	**34.88**	**28.06**	**41.7**	

NOTES: States sorted from high to low ratio of actual/needed hospital beds. ^1^ These three categories are based on whether the actual (column 1) is greater than the adjustment estimate’s lower confidence interval; whether it falls between the two; or whether the actual is below the lower confidence interval.

**Table 3:** Model estimates psychiatric beds (per 100,000 pop.), by U.S. regions, 2018.

REGIONS and Divisions	Psych. Hosp., Beds per 100,000	Model Estimates of Psych Beds, Unadjusted	Model Estimates, Adjusted for Top 10% CMH	95% Confidence Intervals for Adj. Model Estimates		Comparison Actual and Needed (Adjusted) Psychiatric Hospital Beds ^1^
NORTHEAST						
New England	38.3	47.44	38.21	27.18	9.23	Actual = Needed (w/95%)
Middle Atlantic	40.44	42.73	41.12	31.46	50.79	Actual = Needed (w/95%)
Total	39.87	43.98	40.35	30.32	50.37	Actual = Needed (w/95%)
MIDWEST						
East North Central	29.44	31.73	31.38	26.15	36.61	Actual = Needed (w/95%)
West North Central	43.49	29.15	27.88	23.56	32.2	Actual >Needed (w/95%)
Total	33.85	30.93	30.28	25.34	35.23	Actual = Needed (w/95%)
SOUTH						
South Atlantic	40.82	32.21	33.97	28.36	39.58	Actual > Needed (w/95%)
East South Central	31.38	34.49	32.81	25.23	40.39	Actual = Needed (w/95%)
West South Central	37.77	25.53	31.27	25.38	37.16	Actual > Needed (w/95%)
Total	38.38	33.77	32.91	26.91	38.92	Actual = Needed (w/95%)
WEST						
Mountain	72.03	32.21	32.39	26.44	38.35	Actual > Needed (w/95%)
Pacific	31.39	34.49	40.8	32.62	48.97	Actual < Needed (w/95%)
Total	44.28	33.77	38.13	30.66	45.6	Actual = Needed (w/95%)

NOTES: Means are weighted by state population. ^1^ These three categories are based on whether the actual is greater than the adjustment estimate’s lower confidence interval; whether it falls between the confidence intervals; or whether the actual is below the lower confidence interval.

**Table 4:** Comparison of model predictions with prior studies.

Period	Location	Source	Number of Studies	Range	Estimate, per 100,000
1950s	U.K.	Tooth and Brook, 1962 [6]	1	--	180
1960s	Mixed	Bachrach, 1975 [9]	12	40–219	83.8
1970s	Mixed	Bachrach, 1975 [9]	9	20–375	89.4
1980s	U.S.—Mixed states	Goperud,1985 [10]	6	24.1–44.0	33.8
2000s	U.S.	Treatment Advocacy Center, 2008 [18]	1	40–60	50
2010s	U.S.—NC	La, et al., 2016 [19]	1	--	39
2015	World—U.S.	Hudson, 2020 [5]	1	53.5–74.8	64.1
2018	U.S. States	CURRENT STUDY	1	28.1–41.7	34.9

## Data Availability

The data analyzed in this study are available in the publicly available sources described and cited in the Variables and Data Sources section.
